# Acid sphingomyelinase promotes diabetic cardiomyopathy via NADPH oxidase 4 mediated apoptosis

**DOI:** 10.1186/s12933-023-01747-1

**Published:** 2023-02-02

**Authors:** Ruijiao Liu, Tengfei Duan, Li Yu, Yongzhong Tang, Shikun Liu, Chunjiang Wang, Wei-Jin Fang

**Affiliations:** 1 Department of Pharmacy, The Third Xiangya Hospital of Central South University, 138#Tong Zi PoRoad, Changsha, 410013 Hunan China; 2 Department of Ultrasound Diagnosis, The Second Xiangya Hospital of Central South University, Changsha, 410013 Hunan China; 3 Department of Anesthesiology, The Third Xiangya Hospital of Central South University, Changsha, 410013 Hunan China

**Keywords:** Acid sphingomyelinase, Ceramide, Cardiomyopathy, NADPH oxidase 4, Apoptosis

## Abstract

**Background:**

Increased acid sphingomyelinase (ASMase) activity is associated with insulin resistance and cardiac dysfunction. However, the effects of ASMase on diabetic cardiomyopathy (DCM) and the molecular mechanism(s) underlying remain to be elucidated. We here investigated whether ASMase caused DCM through NADPH oxidase 4-mediated apoptosis.

**Methods and results:**

We used pharmacological and genetic approaches coupled with study of murine and cell line samples to reveal the mechanisms initiated by ASMase in diabetic hearts. The protein expression and activity of ASMase were upregulated, meanwhile ceramide accumulation was increased in the myocardium of HFD mice. Inhibition of ASMase with imipramine (20 mg Kg^−1^ d^−1^) or siRNA reduced cardiomyocyte apoptosis, fibrosis, and mitigated cardiac hypertrophy and cardiac dysfunction in HFD mice. The similar effects were observed in cardiomyocytes treated with high glucose (HG, 30 mmol L^−1^) + palmitic acid (PA, 100 μmol L^−1^) or C16 ceramide (CER, 20 μmol L^−1^). Interestingly, the cardioprotective effect of ASMase inhibition was not accompanied by reduced ceramide accumulation, indicating a ceramide-independent manner. The mechanism may involve activated NADPH oxidase 4 (NOX4), increased ROS generation and triggered apoptosis. Suppression of NOX4 with apocynin prevented HG + PA and CER incubation induced *Nppb* and *Myh7* pro-hypertrophic gene expression, ROS production and apoptosis in H9c2 cells. Furthermore, cardiomyocyte-specific ASMase knockout (ASMase^Myh6KO^) restored HFD-induced cardiac dysfunction, remodeling, and apoptosis, whereas NOX4 protein expression was downregulated.

**Conclusions:**

These results demonstrated that HFD-mediated activation of cardiomyocyte ASMase could increase NOX4 expression, which may stimulate oxidative stress, apoptosis, and then cause metabolic cardiomyopathy.

**Supplementary Information:**

The online version contains supplementary material available at 10.1186/s12933-023-01747-1.

## Introduction

The patients with obesity and metabolic syndrome are more likely to exhibit signs of cardiac dysfunction. It has long been recognized that diabetic heart as clinical challenge is associated with increased incidence and risk of heart failure. The diabetes-associated heart failure, which is termed as diabetic cardiomyopathy (DCM), characterized by the left ventricular (LV) diastolic dysfunction in early stage, prior to clinically evident of LV systolic dysfunction, concomitant with the structural, morphological, and metabolic abnormalities in heart [1]. Metabolically, diabetic heart uses more fatty acids as primary energy source than glucose, leading to lipid metabolites accumulation including diacylglycerol and ceramide [2], eventually lipotoxicity [3]. To date, no effective treatment for diabetic cardiomyopathy is available, hence there is a critical need to identify novel therapeutic targets for intervention.

Acid sphingomyelinase (ASMase) is expressed in almost every cell type and is located mainly within the endosomal/lysosomal compartment. ASMase functions as the main enzyme that decomposes the breakdown of the abundant membrane lipid sphingomyelin (SM) to the lipid messenger ceramide (CER) [4]. Lipid modification induced by ASMase can trigger a local shift in membrane properties and influence downstream signaling. Numerous studies in vivo and i*n vitro* have provided strong evidence that ASMase activation and ceramide accumulation were antagonists of insulin signaling and mitochondrial metabolism [5], predicting cardiovascular outcomes in patients [6]. ASMase activity is increased in patients with chronic heart failure and associated with vascular dysfunction and poor long-term outcome [7]. Imipramine and desipramine, which served as functional inhibitors of ASMase, abrogated ceramide-induced apoptosis and protected cardiomyocytes from damage in an ex vivo model [8]. Desipramine also showed the beneficial effect on sepsis-induced cardiac dysfunction at a transcriptional level [9]. A protective effect against diet-induced hyperglycemia and insulin resistance has also been observed in ASMase knockout animals [10]. Similarly, plasma ASMase activity and NOX2-derived peptide were both increased in hypertensive subjects. siRNA-mediated knockdown of ASMase effectively protected against endothelial dysfunction via suppressing NADPH-derived ROS overproduction [11], hinting that ASMase may have regulatory effect on NADPH oxidases. Although all the mentioned above were leading us to speculate that ASMase deficiency may be beneficial for diabetic heart, however, the underlying mechanism of ASMase induced structural and functional abnormalities under metabolic disorders remains incompletely understood.

Thus, we here investigated the role of ASMase in the regulation of metabolic cardiomyopathy in both type 2 diabetes mice model and the high-fat diet (HFD) consumption mice model. The purpose of present study was to find out: (1) whether ASMase affects the development of diabetic cardioymyopathy; (2) whether ASMase plays an essential role in regulation of NADPH oxidases; (3) whether NADPH oxidases is required for ASMase-mediated apoptosis and cardiac dysfunction.

## Materials and methods

### Animals and high-fat diet feeding

All animal experiments were performed according to the Guidelines of Animal Experiments from Committee of Medical Ethics at the National Health Department of China and were approved by Central South University. Specific pathogen-free (SPF) male C57BL/6 mice with body weight of 20 ± 2 g aged 6 weeks were purchased from Department of laboratory Animals of Central South University (Changsha, China). Upon arrival, the mice were acclimatized in separate cages for 1 week with water and food available ad libitum. A total of 24 mice were randomized to 4 groups equally. The mice were fed a high-fat diet (HFD, 60%kcal, D12492) for 16 weeks and then subjected to Imipramine hydrochloride (Imi, 20 mg Kg^−1^ d^−1^ by gavage, dissolved in 0.9% NaCl) or vehicle for another 4 weeks concurrent with continuous HFD feeding. The control mice were fed a standard chow diet (chow, 10%kcal, D12450B).

ASMase^flox/flox^ (ASMase^fl/fl^) mice and myosin heavy chain 6(Myh6)-Cre mice were purchased from Cyagen Biosciences Inc, Suzhou, China. ASMase^fl/fl^ mice were crossed with Myh6-Cre mice to obtain ASMase^Myh6KO^ mice through excising specifically exon 2 of the ASMase gene in cardiomyocytes. The ASMase protein expression levels were significantly decreased in hearts of ASMase^Myh6KO^ mice relative to ASMase^fl/fl^ mice (Additional file 1: Figure S1).

### H9c2 cell cultures and treatment

H9c2 cell is a subclone of the original clonal cell line derived from embryonic BD1X rat heart tissue and therefore was used to cardiac myocytes mimic for exploring underlying mechanisms in the present study. H9c2 cells were obtained from the Institute of Biochemistry and Cell Biology (Shanghai Institute for Biological Science, the Chinese Academy of Sciences, Shanghai, China) and cultured in Dulbecco’s modified Eagle’s medium (DMEM, Gibco, Eggenstein, Germany) containing 5.5 mmol/L D-glucose supplemented with 10% FBS, 100 U/ml penicillin and 100 mg/ml streptomycin at 37 °C in a humidified atmosphere of 5% CO2. The cells were divided into the following groups after they reached 70% confluency: (1) the high glucose plus palmitic acid group (HG + PA) in which cardiomyocytes were incubated with DMEM medium containing 30 mM glucose and 100 μM palmitic acid; (2) the ceramide group (CER) in which 20 μM C16 ceramide was added to the DMEM medium; (3) the imipramine group (Imi) in which 50 μM imipramine was added to the DMEM medium; (4) the imi + HG + PA group in which 50 μM imipramine was added to DMEM medium with 30 mM glucose and 100 μM palmitic acid; and (5) the Imi + CER group in which 50 μM imipramine was added to the DMEM medium with 20 μM C16 ceramide; (6)the apocynin group (Apo) in which 100 μM apocynin was added to the DMEM medium; (7) the Apo + HG + PA group in which 100 μM apocynin was added to the DMEM medium with 30 mM glucose and 100 μM palmitic acid; (8) the Apo + CER group in which 100 μM apocynin was added to the DMEM medium with 20 μM C16 ceramide. All cell cultures were maintained at 37 °C in a humidified incubator containing 5% CO_2_ for 24 h and then harvested and stored at − 80 °C for further analysis.

### siRNA transfection

The H9c2 cardiomyocytes were seeded in 30,000 cells per square cm in six-well plates or 96-well plates, and transfection began at a cell density of 30%-50% by using the commercially available siRNA transfection reagent (RIBOBIO, Cat. No. C10511-05). H9c2 cells were transfected with 100 nM of Smpd1small interfering RNA (RIBOBIO, siRNA-Smpd1, 5′-GCTACCGAGTTTACCAAAT-3′) or negative control (NC, 5′-UUCUCCGAACGUGUCACGUTT-3′) according to the manufacturer’s protocol. After 24 h transfection, cells were treated with specific concentrations of drugs, followed by a total incubation for another 24 h.

### Echocardiography

Echocardiography was performed by using Visual Sonics Vevo 2100 equipment with a 30 MHz transducer at 16 weeks after high fat diet feeding. In brief, the anesthetized mice with 3% isoflurane were placed on a homothermic platform with embedded electrocardiograph. Parasternal short-axis M-mode echocardiographic images were obtained at papillary muscles level. The following parameters were recorded to reflect cardiac function: LV ejection fraction (LVEF %), LV shortening fraction (LVFS %), cardiac output, LV anterior wall thickness at systole (LVAW;s), LV anterior wall thickness at diastole (LVAW;d), LV posterior wall thickness at systole (LVPW;s), LV posterior wall thickness at diastole (LVPW;d), LV volume at systole(Volume;s), LV volume at diastole (Volume;d), LV internal dimension at systole (LVID;s); LV internal dimension at diastole (LVID;d), the ratio of early to late diastolic mitral flow velocities (E/A).

### Reactive oxygen species (ROS) generation determination

Oxidative stress was evidenced by ROS generation through 2’-,7’-dichloro-fluorescin diacetate (DCFH-DA) staining. Briefly, cells were plated on 96-well plate, allowed to attach overnight, and then treated with or without 30 mM glucose and 100 μM palmitic acid, 20 μM C16 ceramide, 50 μM imipramine, 100 μM apocynin. All procedures were according to the manufacturer's instructions. The DCFH-DA-fluorescence intensities (representing the intracellular ROS levels) were determined using High Content Screening System (PerkinElmer, Massachusetts, USA) and the data were analyzed using Operetta^®^ High Content Imaging System (PerkinElmer, Massachusetts, USA).

### Actin staining

Actin staining was performed to measure the size of H9c2 cardiomyocytes according to manufacturer’s protocol. Briefly, H9c2 cells were cultured in 3599 cell culture plates, then were stained after 24 h of additive treatment. Cells were first fixed with 4% paraformaldehyde for 15 min, washing with PBS containing 0.1% Triton X-100. Actin were stained with Actin-Tracker Red-Rhodamine (Beyotime, China) and nuclei was stained with 4′,6-diamidino-2-phenylindole (DAPI, Beyotime, China). The whole staining progress was conducted under dark at room temperature. The cytoskeleton of the cardiomyocytes was visualized, and images were captured by the Operetta High Content imaging system (Thermo Fisher Scientific, Perkin Elmer, USA) with 540 nm excitation wavelength, 565 nm emission wavelength.

### Myocardial apoptosis assessment

H9c2 cells were treated as described above and apoptotic cells were assessed by Terminal Deoxynucleotidyl Transferase-Mediated dUTP Nick End Labeling (TUNEL) Staining with one-step TUNEL apoptosis detection kit (Beyotime, China) in accordance with the manufacturer’s protocol. In brief, H9c2 cells were fixed with 4% paraformaldehyde solution for 30 min, washed twice with PBS, and the cells were permeabilized using an immunostaining permeabilization solution. Then, the cells were incubated in TUNEL staining solution at 37 °C for 2 h and subsequently stained with DAPI solution. Finally, the cells were washed three times with PBS and observed under a high-connotation imaging system (Thermo Fisher Scientific, Perkin Elmer, USA). For TUNEL detection, the excitation and emission wavelengths were set to 460 nm to 490 nm and 500 nm to 550 nm, respectively.

The apoptosis in cardiac tissues was further evaluated using TUNEL assay (Servicebio, Wuhan, China). Briefly, the sections were incubated with xylene and ethanol respectively to deparaffinize and rehydrate. Then, the tissues were added proteinase K working solution and incubated at 37 °C for 25 min for antigen retrieval. Next, the permeabilize working solution (0.1% Triton) was added and incubated at room temperature for 20 min for permeabilization. Subsequently, the sections were incubated with TUNEL reaction solution at 37 °C for 2 h under dark. DAPI solution was used for nucleus staining at room temperature for 10 min. The images were collected under NIKON ECLIPSE C1 confocal microscope (Nikon, Japan).

### Hematoxylin & eosin (HE), MASSON and wheat germ agglutinin (WGA) staining

Hearts fixed in 4% paraformaldehyde were dehydrated in ethanol, cleared in xylene, and embedded in paraffin, respectively. The paraffin-embedded heart tissues were cut into 5 μm sections on a microtome. After deparaffinized, the sections were stained with hematoxylin and eosin (H&E) and MASSON. The sections were viewed and pictured under a light microscope. For the wheat germ agglutinin (WGA) staining, the 5 μm paraffin-embedded sections of hearts were prepared and stained with WGA following the instruction of manufacture’ protocol. The sections were viewed and photographed under a fluorescence microscope. The green fluorescence indicates the cell membrane, and the cross-section area of cardiomyocyte was measured by Image J software. Then, the data was normalized and presented.

### Reverse transcription quantitative polymerase chain reaction (RT-qPCR)

The total RNA was isolated from tissues or cells using RNA-easy Isolation Reagent (R701-01, Vazyme). The mRNA was reversely transcribed into cDNA by using PrimeScript reverse transcriptase kit (11141ES60, Yeasen Biotechnology, Shanghai, China). RT-qPCR was conducted according to the instructions of TaqMan Gene Expression Assays protocol (Applied Biosystems, Foster City, CA, USA). Three replicates were set for RT-qPCR. The primer sequences (Additional file 2: Table S1) were designed based on the NCBI (https://www.ncbi.nlm.nih.gov/tools/primer-blast/). The 2^−ΔΔCt^ method was used to quantify the relative expression of target genes.

### Western blot analysis

The protein was extracted from mouse heart tissues and H9c2 cardiomyocytes using protease inhibitor-containing radio immunoprecipitation assay (RIPA) lysis buffer (KGP702, Keygen Biotech, Jiangsu, China). The protein concentration was detected by bicinchoninic acid (BCA) kit (KGP902, Keygen Biotech, Jiangsu, China). The protein was qualified according to different concentrations. The protein was separated by sodium dodecyl sulfate–polyacrylamide gel electrophoresis (SDS-PAGE), electrotransferred onto a polyvinylidene fluoride (PVDF) membrane (200 mA, 75 min). The membrane was blocked with 5% skim milk for 1 h, and then incubated with the prepared primary antibodies ASMase (3687S, 1:1000, Cell Signaling Technology), NOX2 (19013-1-AP, 1:3000, Proteintech Group), NOX4 (14347-1-AP, 1:3000, Proteintech Group), Cleaved-caspase-3 (9662S, 1:1000, Cell Signaling Technology), 4- Hydroxynonenal (ab46545, 1:3000, Abcam) and β-actin (BS6007M, 1:6000, Bioworlde) at 4 °C overnight. Subsequently, the membrane was washed by Tris-buffered saline with Tween (TBST) 4 times, 5 min per time, followed by 1 h incubation with the horseradish peroxidase (HRP)-marked secondary antibody goat anti-rabbit IgG (511203, ZENBIO) or goat anti-mouse IgG (511103, ZENBIO) diluted at 1:10000. After TBST rinses, the membrane was developed with luminescent liquid. The results were analyzed by the ImageJ software (National Institutes of Health, Bethesda, Maryland, USA). The relative protein expression was expressed as the ratio of the gray value of protein to be tested to that of internal reference (GAPDH).

### Statistical analyses

All values are shown as the mean ± SEM. Data were analyzed by using GraphPad Prism version 9.0 software (San Diego, CA). One-way analysis of variance (ANOVA) was performed for multiple comparisons. *P* < 0.05 were considered as statistically significant.

## Results

### HFD-induced diabetic cardiomyopathy is blunted by ASMase inhibitor

**Fig. 1:**
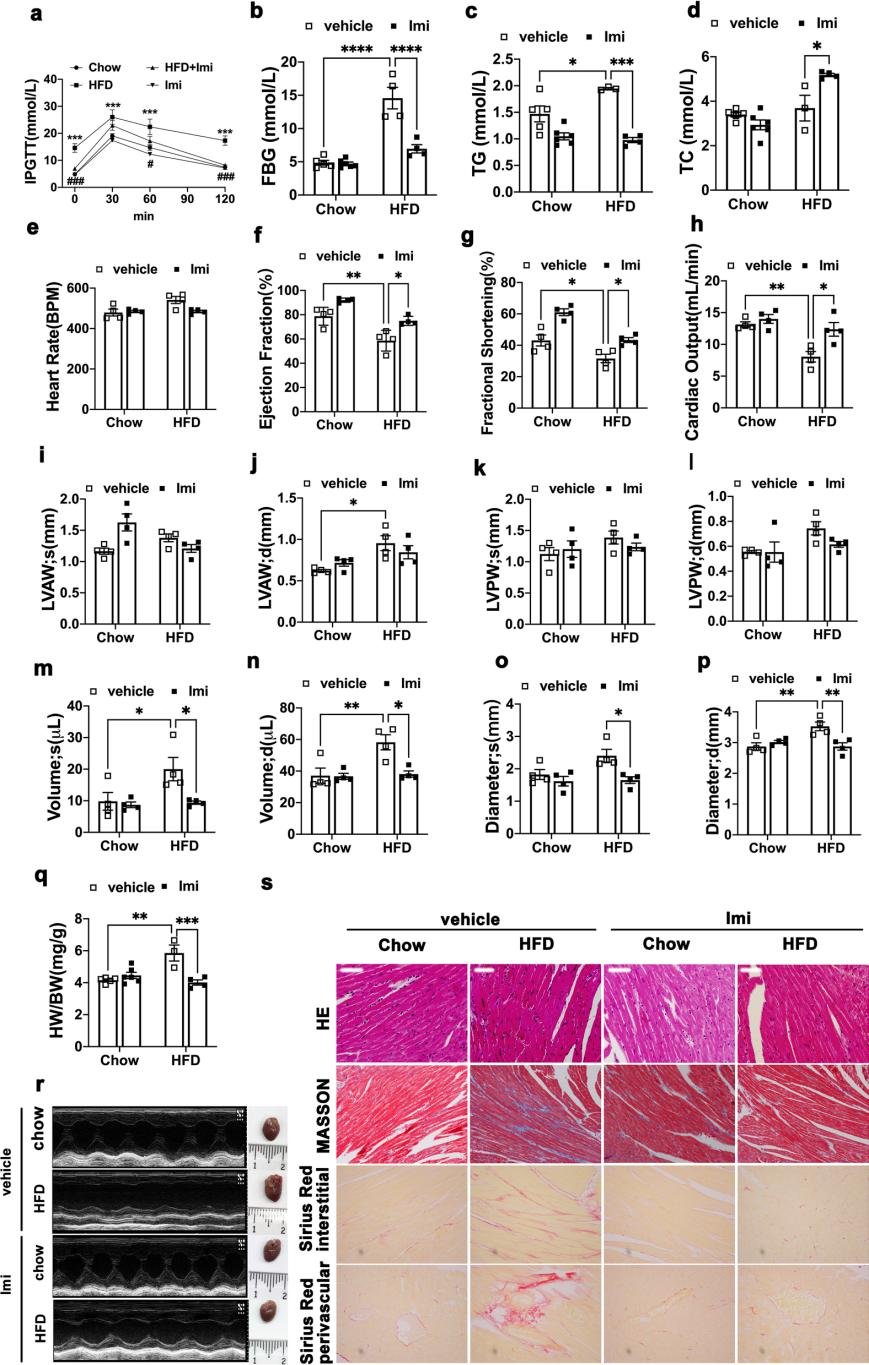
HFD-induced cardiac remodeling is blunted by ASMase inhibitor. C57B/L6 mice were treated with high fat-diet for 6 weeks and then were given imipramine, an ASMase inhibitor for 4 weeks. **a** Glucose tolerance test was performed by intraperitoneal injection of glucose. **b**–**d** Fasting blood glucose levels, total triglyceride levels and cholesterol levels were detected by biochemical methods. **e**–**p** Echocardiographic parameters were recorded. In order, they are heart rate, ejection fraction(EF %), fraction shortening(FS %), cardiac output, left ventricular anterior wall thickness at systole (LVAW; s), left ventricular anterior wall thickness at diastole (LVAW; d), left ventricular posterior wall thickness at systole (LVPW; s), left ventricular posterior wall thickness at diastole (LVPW; d), left ventricular volume at systole(Volume; s), left ventricular volume at diastole (Volume; d), left ventricular internal dimension at systole (LVID; s); left ventricular internal dimension at diastole (LVID; d). **q** The heart weight to body weight was calculated. **r** Echocardiography images. **s** Myocardial hypertrophy and fibrosis were assessed by H&E, MASSON trichrome and Sirius Red staining (Scale bar:100 μm). Data were presented as Mean ± SEM. n = 5. ^*^P < 0.05, ^**^P < 0.01, ^***^P < 0.001, ^****^P < 0.0001

To explore whether ASMase was correlated with cardiomyopathy in animal models of diabetes, we induced type 2 diabetes (T2D) in C57BL/6 mice, which were fed on high-fat diet for 16 weeks. These mice gained weight faster than control mice fed on chow, had higher plasma fasting glucose levels (Fig. 1b), impaired glucose tolerance (Fig. 1a), and hypertriglyceridemia (Fig. 1c). However, plasma cholesterol levels were not significantly different between HFD and Chow mice without imipramine (Fig. 1d). To our surprise, imipramine significantly increased plasma cholesterol level in HFD mice.

Heart weight was increased in HFD mice compared with Chow mice (Fig. 1Q) along with a significantly increase in the cross-sectional area of the cardiomyocytes (Fig. 1S). Furthermore, the echocardiographic assessment showed that LV dimension volume at systole (Volume; s, Fig. 1m) and diastole(Volume; d, Fig. 1n), LV dimension diameters at systole (Diameters; s, Fig. 1o) were markedly increased in the HFD treated mice, while left ventricular anterior wall and posterior wall thickness (LVAW and LVPW, Fig. 1i–l) showed an increasing trend. Cardiac output (CO, Fig. 1h) was decreased in HFD mice, even though ejection fraction (EF%, Fig. 1f) and fractional shortening (FS%, Fig. 1g) just showed a trend of reduction. What’s more, morphometric and histological analysis were performed at 16 weeks after HFD and showed disordered arrangement of myocardial fibers and a significant deposition of fibrotic tissue in the left ventricle of HFD animals. Staining of heart tissues with Sirius Red and Masson’s Trichrome indicated excessive fibrosis in HFD mice (Fig. 1s). Treatment with imipramine reverted all above parameters, CO and fibrosis, back to values like those observed in chow animals.

### Cardiac ASMase is activated to promote NADPH oxidases activity and apoptosis in HFD mice

**Fig. 2:**
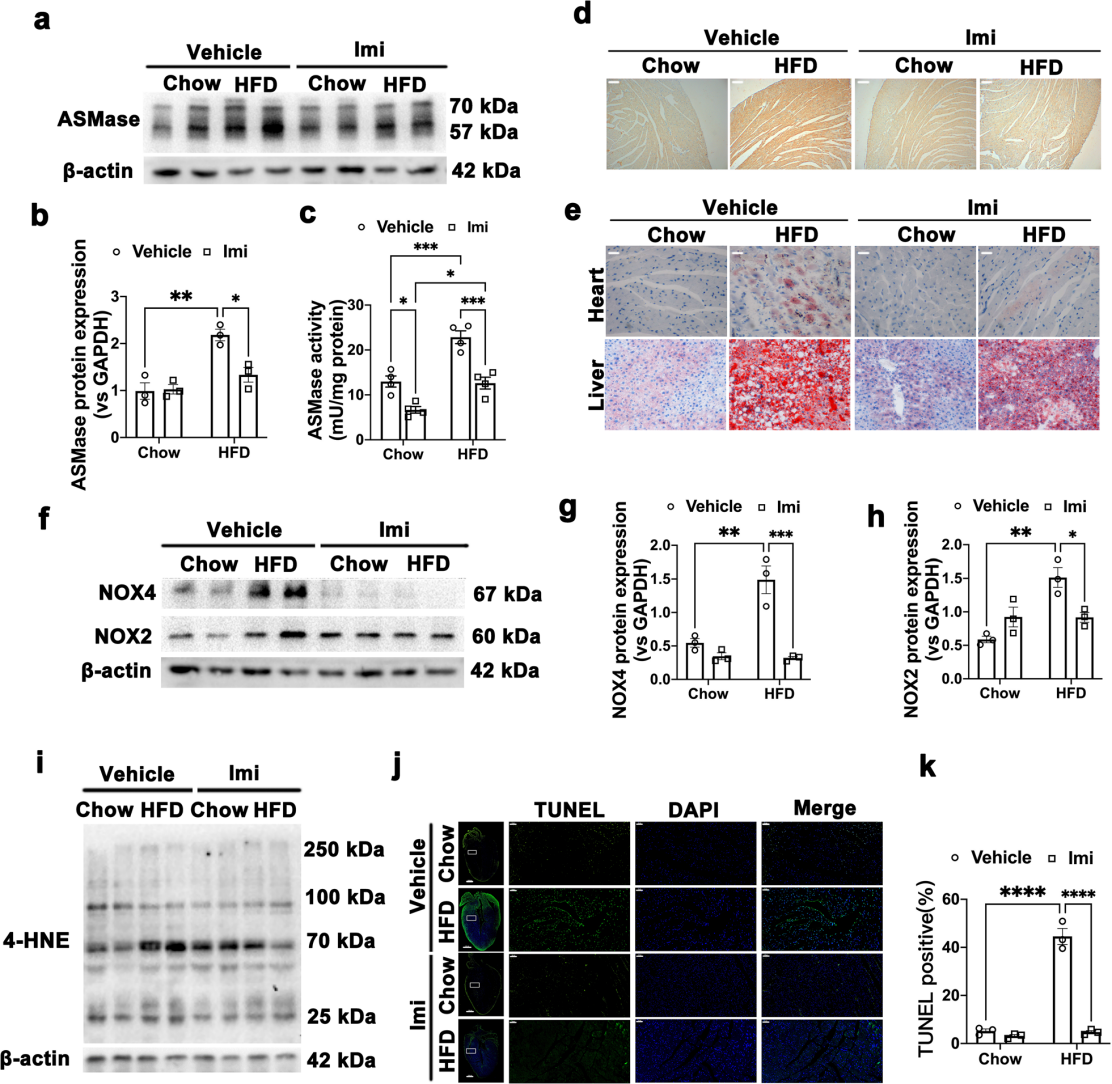
Cardiac ASMase promotes NADPH oxidases and apoptosis in HFD mice. **a** Representative Western blot images of ASMase expression in the myocardium of HFD-induced mice with or without imipramine. **b** Quantitative analysis of ASMase expression, β-actin was used as a loading control. **c** ASMase activity was detected by assay kit. **d** Representative immumohistochemical staining of ceramide (scale bar: 100 μm). **e** Representative images of Oil Red staining in hearts and livers from four groups of mice (scale bar: 100 μm). **f**–**i** Representative Western blot images and quantitative analysis of NOX4, NOX2 and 4-HNE protein expressions in hearts of four groups. β-actin was used as a loading control. **j** Representative fluorescence images of TUNEL staining (left scale bar: 1000 μm; right scale bar: 50 μm). **k** Quantitative analysis of TUNEL-positive cells in myocardial tissues. Data were presented as mean ± SEM, n = 3. ^*^P < 0.05, ^**^P < 0.01, ^***^P < 0.001, ^****^P < 0.0001

We have shown that multiple abnormal parameters involving lipid metabolism and cardiac function during HFD treatment are ablated by ASMase inhibitor, imipramine. We next asked whether ASMase and its metabolites, ceramide (CER) were served as direct causative roles to trigger DCM and regulate downstream molecular mechanism. We observed that protein expression of cardiac ASMase was significantly increased after 16 weeks HFD feeding, concomitant with highly elevated ASMase activity (Fig. 2a–c). As expected, activated ASMase dramatically motivated ceramide (CER) accumulation in hearts of HFD animals (Fig. 2d). Meanwhile, tissue sections of HFD hearts revealed obvious lipid accumulation, evidenced by Oil Red O staining, compared with chow hearts (Fig. 2e), consistent with changes of plasma lipid parameters. In contrast, imipramine administration clearly diminished cardiac ASMase activity and protected against the gain of ceramide and lipid content in HFD-treated animals. These data support the notion that attenuation of cardiac dysfunction and fibrosis in imipramine treated HFD mice was due, in part, to blockade of ASMase activity.

Oxidative stress caused by ceramide accumulation have demonstrated to implicate in obesity and diabetes-associated cardiac dysfunction pathogenesis. However, direct in vivo evidence of ASMase participation is missing. To address this question, we examined NADPH oxidase enzymes (NOXs), whose primary function is to generate reactive oxygen species (ROS), and its oxidative product, 4-hydroxynonenal (4-HNE) contents. The HFD mice exhibited higher NOX2 and NOX4 protein expressions. Antibody against 4-HNE representing HNE modified proteins quantified as total 4-HNE levels were also increased significantly compared with chow mice, which were reversed by ASMase inhibitor (Fig. 2f–i). Furthermore, apoptosis occurrence was examined by TUNEL assay and high cleaved caspase-3 expression, which also reversed by ASMase inhibitor, imipramine (Fig. 2j–k). Together, this analysis reveals that ASMase is activated and may promotes HFD-induced cardiomyopathy via NADPH oxidases-mediated oxidative stress and apoptosis.

### High glucose, palmitate acid and ceramide positively activate ASMase and induce oxidative stress and apoptosis in H9c2 cells

**Fig. 3:**
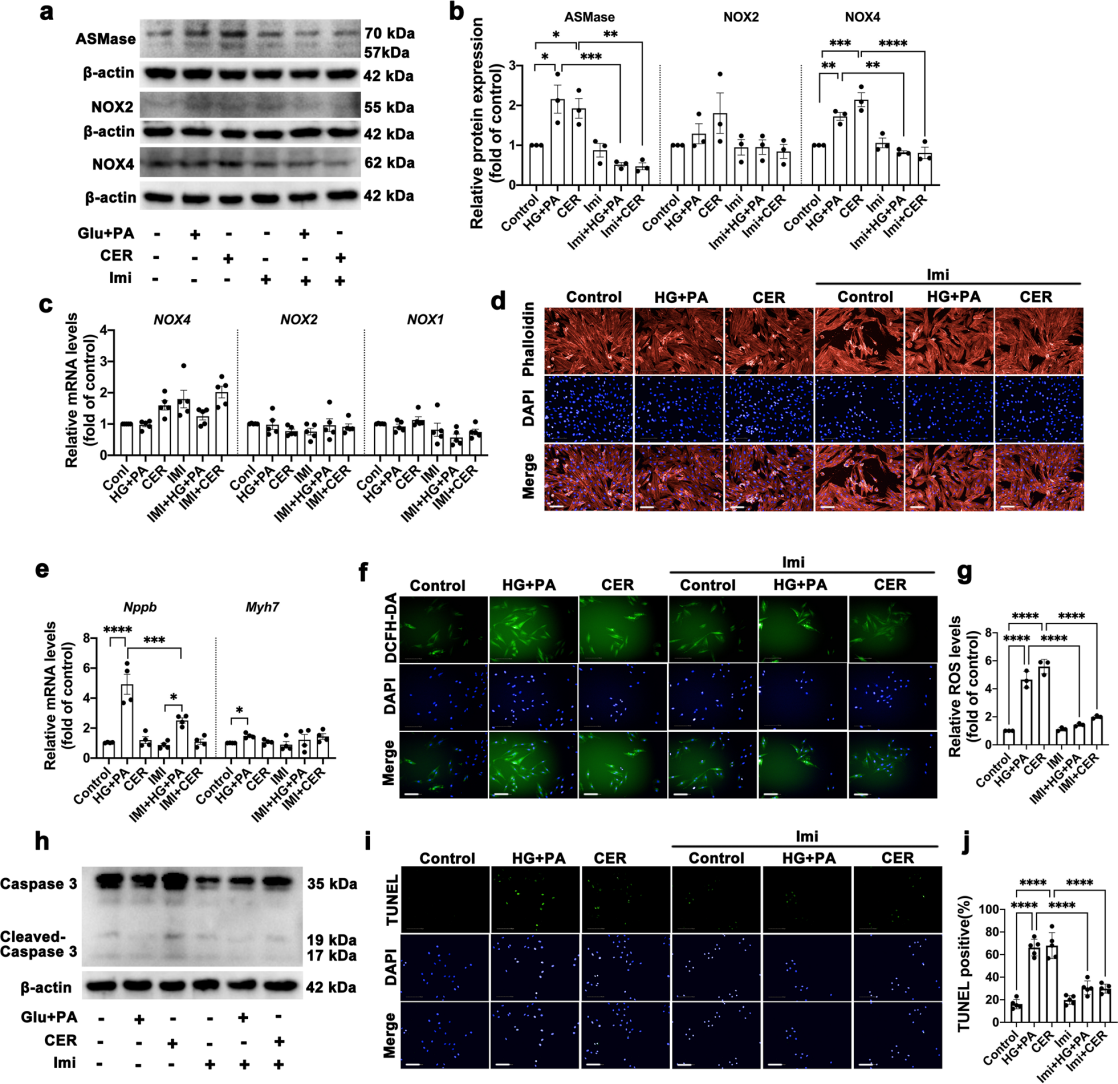
High glucose, palmitate acid and ceramide positively activate ASMase and induce oxidative stress and apoptosis in H9c2 cells. **a** Representative Western blot images of protein expression of ASMase, NOX2, and NOX4 in six groups of H9c2 cells treated with vehicle, glucose + PA, ceramide, imipramine, imipramine + glucose + PA, imipramine + ceramide, respectively. β-actin was used as a loading control. **b** Quantitative analysis of ASMase, NOX2, and NOX4 expression in H9c2 cells treated as above. Three independent experiments were performed to calculate the means. **c** Quantitative analysis of NOX1, NOX2 and NOX4 mRNA expression by qPCR in H9c2 cardiomyocytes. Five independent experiments were performed to calculate the means. **d** Representative fluorescence images of rhodamine-phalloidin staining to visualize cell size, scale bar: 100 μm. **e** Quantitative analysis of pro-hypertrophic markers, *Nppb* and *Myh7* mRNA expression by qPCR in H9c2 cardiomyocytes. Five independent experiments were performed to calculate the means. **f**–**g** Representative fluorescence images and quantitative analysis of DCFH-DA staining for ROS measurement in H9c2 cells, scale bar:100 μm. Three independent experiments were performed to calculate the means. **h** Representative Western blot images of protein expression of cleaved caspase-3, β-actin was used as a loading control. **i**–**j** Representative fluorescence images and quantitative analysis of TUNEL staining, scale bar: 100 μm. Five independent experiments were performed to calculate the means. Data were presented as mean ± SEM, n = 3. ^*^P < 0.05, ^**^P < 0.01, ^***^P < 0.001, ^****^P < 0.0001

We next sought to delineate whether HFD-induced cardiomyopathy was directly caused by ASMase. H9c2 cardiomyocytes were treated with high glucose and palmitate acid (HG + PA) or exogeneous CER for 24 h to mimic the hyperglycemia and hyperlipemia in vivo. We found that both HG + PA and exogeneous CER resulted in obvious upregulation of ASMase protein expressions (Fig. 3a–b). HG + PA treatment remarkably caused cardiomyocytes hypertrophy as evidenced by increased *Nppb* and *Myh7* gene expressions. However, cardiac hypertrophy was not caused by addition of CER (Fig. 3d–e). Imipramine prevented against HG + PA and CER induced ASMase protein expression and restored cardiac hypertrophy.

Next, we went on to assess whether HG + PA and CER mediated ASMase activation was involved in NADPH oxidases activation and apoptosis. We verified that HG + PA and CER treatments led to approximately 2 or threefold of NOX4 protein expressions (Fig. 3a–b), while NOX2 expression was not affected. What is noteworthy is that mRNA levels of NOXs were not changed by HG + PA and CER administration (Fig. 3c). ROS production, indicated by fluorescence, was sharply elevated 12 h after treatment with HG + PA and CER (Fig. 3f–g). These effects were also antagonized by imipramine. Oxidative stress can induce cell apoptosis. Therefore, TUNEL assay was performed. As anticipated, HG + PA and CER caused cell apoptosis, which is consistent with the observation in upregulation of cleaved caspase-3 protein (Fig. 3h–j). Application of imipramine restored cell viability and alleviated cleaved caspase-3 expressions, which were served as both oxidative damage and apoptosis indicators. Together, these data demonstrate that high glucose, palmitate acid and ceramide can activate ASMase and induce oxidative stress and apoptosis in H9c2 cells.

### ASMase knockdown dampens high glucose, palmitate acid and ceramide induced oxidative stress and apoptosis in H9c2 cells

**Fig. 4:**
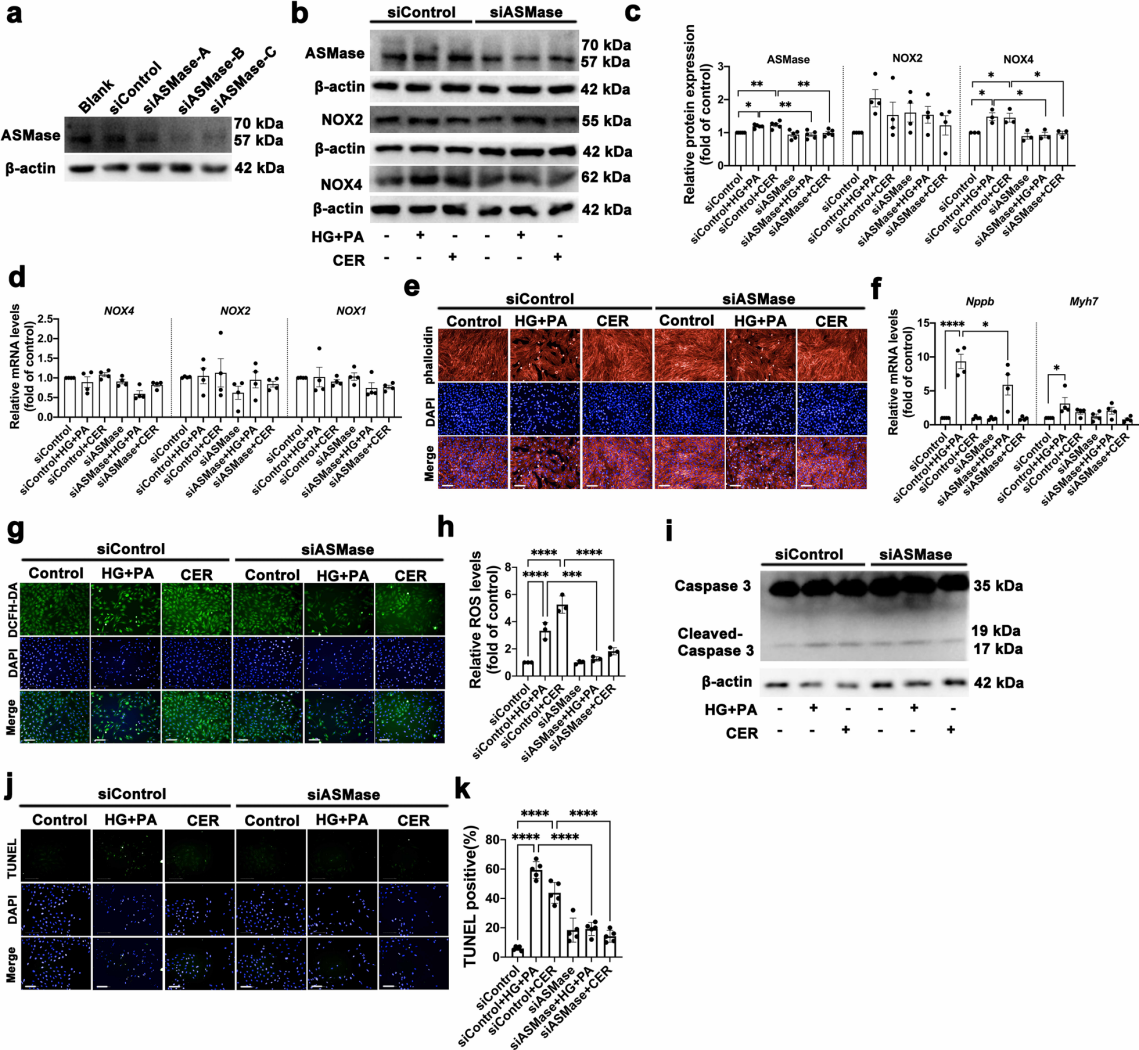
ASMase knockdown dampens high glucose, palmitate acid and ceramide induced oxidative stress and apoptosis in H9c2 cells. **a** Representative Western blot images of ASMaseexpression when H9c2 cells were posed to vehicle or pretransfected with siRNA *ASMase* (si-ASMase), scrambled siRNA (si-control) was used as control (n = 5). **b**–**c** Representative Western blot and quantitative analysis images of ASMase, NOX2 and NOX4 expression when H9c2 cells were treated with vehicle, glucose + PA, ceramide, siASMase, siASMase + glucose + PA, siASMase + ceramide, respectively. β-actin was used as a loading control. Three to five independent experiments were performed to calculate the means. **d** Quantitative analysis of NOX1, NOX2 and NOX4 mRNA expression by qPCR in H9c2 cardiomyocytes after transfected by si-ASMase. Four independent experiments were performed to calculate the means. **e** Representative fluorescence images of rhodamine-phalloidin staining to visualize cell size, scale bar:100 μm. **f** Quantitative analysis of pro-hypertrophic markers, *Nppb* and *Myh7* mRNA expression by qPCR in H9c2 cardiomyocytes. Four independent experiments were performed to calculate the means. **g**–**h** Representative fluorescence images and quantitative analysis of DCFH-DA staining for ROS measurement in H9c2 cells, scale bar:100 μm. Three independent experiments were performed to calculate the means. **i** Representative Western blot images of protein expression of cleaved caspase-3, β-actin was used as a loading control. **j**–**k** Representative fluorescence images and quantitative analysis of TUNEL staining, scale bar:100 μm. Five independent experiments were performed to calculate the means. Data were presented as mean ± SEM, n = 3. ^*^P < 0.05, ^**^P < 0.01, ^***^P < 0.001, ^****^P < 0.0001

We next tested whether HG + PA and CER-triggered oxidative stress and apoptosis was ASMase-dependent. ASMase knocked down in H9c2 cells was performed by using siRNA. Three different siRNA sequences were designed and used in present study. As shown in Fig. 4a, sequence C caused a marked decline in ASMase protein expression and was used for the following experiments. Relative to the vehicle group, the protein expression of NOX4 was significantly increased in the HG + PA or CER group, which was reversed in the ASMase-siRNA group. However, NOX2 expression level had no change regardless of HG + PA or CER treatment with or without ASMase-siRNA (Fig. 4b–c). Unexpectedly, the transcriptional levels of NOX4, NOX2 and NOX1 were not altered by HG + PA and CER, nor was it altered by ASMase-siRNA treatment (Fig. 4d), suggesting a crucial role of ASMase in regulating NOX4 protein expression.

We also tested the potential for ASMase to modulate ROS generation and apoptosis in vitro. Incubation with HG + PA or CER augmented the ROS generation and cell apoptosis, as well as marked elevated cleaved-caspase-3 expression, whereas in the presence of ASMase-siRNA, these responses were partially ameliorated (Fig. 4g–k). Intriguingly, although we observed a similar effect in both HG + PA and CER on H9c2 cells, it was worth noting that CER could not induce cardiomyocyte hypertrophy, which was different from HG + PA. These results suggested that oxidative stress, cardiac hypertrophy, and apoptosis were enhanced by high glucose, palmitate acid or ceramide treatment and restored by ASMase knockdown.

### NOX4 activation is required for ASMase-mediated cardiac hypertrophy and apoptosis

**Fig. 5:**
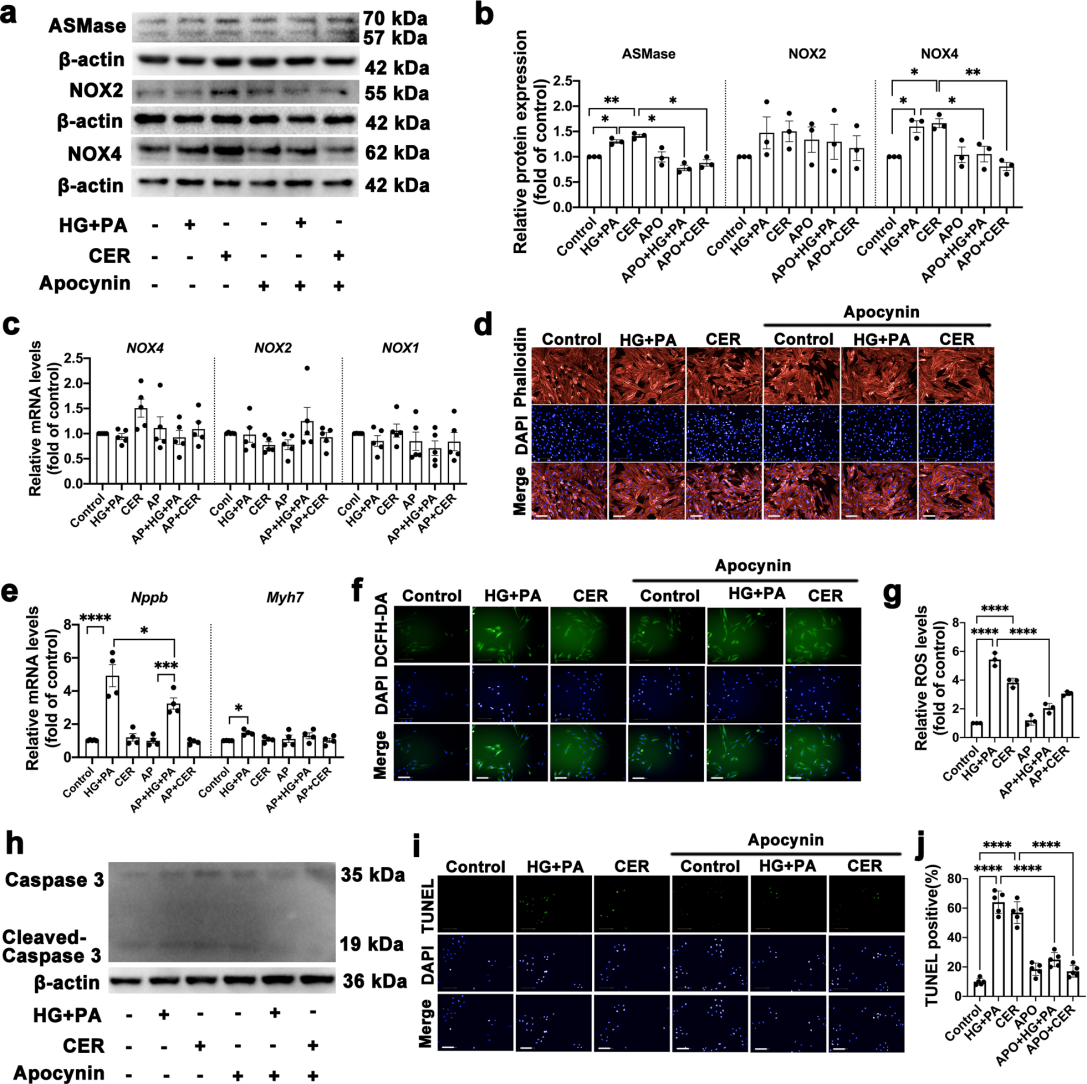
NOX4 activation is required for ASMase-mediated cardiac hypertrophy and apoptosis. **a**–**b** Representative Western blot images and quantitative analysis of protein expression of ASMase, NOX2, and NOX4 in six groups of H9c2 cells treated with vehicle, glucose + PA, ceramide, apocynin, apocynin + glucose + PA, apocynin + ceramide, respectively. β-actin was used as a loading control. Three independent experiments were performed to calculate the means. **c** Quantitative analysis of NOX1, NOX2 and NOX4 mRNA expression by qPCR in H9c2 cardiomyocytes. Five independent experiments were performed to calculate the means. **d** Representative fluorescence images of rhodamine-phalloidin staining to visualize cell size, scale bar:100 μm. **e** Quantitative analysis of pro-hypertrophic markers, *Nppb* and *Myh7* mRNA expression by qPCR in H9c2 cardiomyocytes. Five independent experiments were performed to calculate the means. **f**–**g** Representative fluorescence images and quantitative analysis of DCFH-DA staining for ROS measurement in H9c2 cells, scale bar:100 μm. Three independent experiments were performed to calculate the means. **h** Representative Western blot images of protein expression of cleaved caspase-3, β-actin was used as a loading control. **i**–**j** Representative fluorescence images and quantitative analysis of TUNEL staining, scale bar:100 μm. Five independent experiments were performed to calculate the means. Data were presented as mean ± SEM, n = 3. ^*^P < 0.05, ^**^P < 0.01, ^***^P < 0.001, ^****^P < 0.0001

We next investigated whether NADPH oxidases were necessary for ASMase-mediated oxidative stress, apoptosis, and hypertrophic growth of cardiomyocytes. We took approach to suppress NADPH oxidases by apocynin, a commonly used inhibitor for enzymes. The H9c2 cells were pre-incubated with 100μMapocynin for 30 min and then were exposed to HG + PA and CER for 24 h respectively. The protein expressions of ASMase and NOX4 were substantially promoted by HG + PA and CER as noted above. Apocynin, served as an NADPH oxidases inhibitor, significantly suppressed NOX 4 expression. However, apocynin did not affect ASMase protein expression (Fig. 5a–b). Meanwhile, we found no statistical difference in mRNA levels of NADPH oxidases 4, 2 and 1(Fig. 5c). Furthermore, a decline in ROS generation and apoptosis was observed when NOX4 activity were abolished by addition of apocynin (Fig. 5f–j). Apocynin also exerted an anti-hypertrophy effect as shown by inhibition of Nppb gene expression that caused by HG + PA (Fig. 5d–e). These data enabled us to visualize that NOX4 functioned as a downstream target of ASMase and was required for ASMase. Inhibition NADPH oxidases would rescue cardiac function under ASMase activation.

### *ASMase*^*Myh6KO*^* mice are protected from HFD-induced cardiomyopathy*

**Fig. 6:**
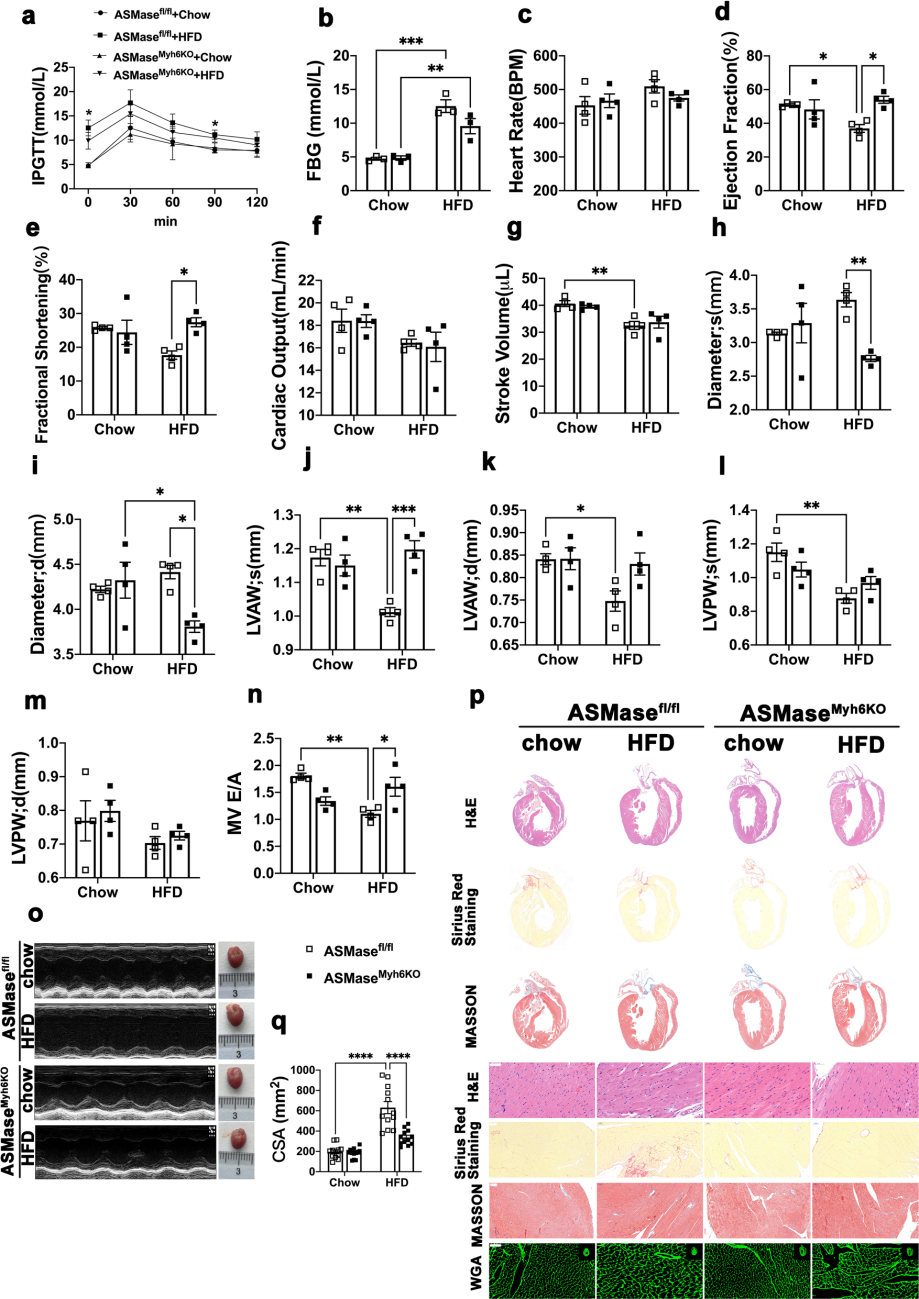
ASMase^Myh6KO^ mice are protected from HFD-induced cardiomyopathy. ASMase^Myh6KO^ mice and littermate’s wildtype mice were treated with high fat-diet for 16 weeks. **a** Glucose tolerance test was performed by intraperitoneal injection of glucose. **b** Fasting blood glucose levels was detected by biochemical methods. **c**–**n** Echocardiographic parameters were recorded: heart rate, ejection fraction(EF %), fraction shortening(FS%), cardiac output, stroke volume, left ventricular internal dimension at systole (LVID;s); left ventricular internal dimension at diastole (LVID;d), left ventricular anterior wall thickness at systole (LVAW;s), left ventricular anterior wall thickness at diastole (LVAW;d), left ventricular posterior wall thickness at systole (LVPW;s), left ventricular posterior wall thickness at diastole (LVPW;d), the ratio of early to late diastolic mitral flow velocities (E/A). **o** M-mode echocardiogram showing left ventricular dimensions. **p** Myocardial hypertrophy and fibrosis were assessed by H&E, MASSON trichrome, Sirius Red staining and wheat germ agglutinin (WGA)staining (Scale bar:50 μm). **q** Cross section area (CSA) of cardiomyocytes size was calculated according to WGA staining. Data were presented as Mean ± SEM. n = 4. ^*^P < 0.05, ^**^P < 0.01, ^***^P < 0.001, ^****^P < 0.0001

To explore the therapeutic potential of ASMase in HFD-induced cardiomyopathy and exclude the systemic influence of drug, we generated ASMase^Myh6KO^ mice by CRISPR/Cas9 genome-editing technology confirmed ASMase depletion in vivo. ASMase^Myh6KO^ mice had no significant effects on blood glucose levels and glucose tolerance (Fig. 6a–b). However, results from echocardiographic measurements showed that LVEF, fractional shortening (FS), the ratio of the early to late diastolic mitral inflow velocities (E/A) were all dramatically increased whereas LVESD and LVEDD were decreased in ASMase^Myh6KO^ + HFD mice compared to those in HFD group at end of 20 weeks (Fig. 6c–n). Morphologically, HFD mice exhibited significantly myocardial disarray under H&E staining, which is reversed by ASMase depletion. Heart sections were also stained with Masson to evaluate the degrees of fibrosis. The results revealed that cardiac fibrosis was improved in ASMase deficiency mice compared with control. Cardiac sections stained with wheat germ agglutinin (WGA) showed that ASMase deficiency decreased cardiomyocyte cross-sectional area (Fig. 6p–q). These results suggested that ASMase deficiency single attenuated HFD-induced cardiac dysfunction, hypertrophy, and fibrosis. These improvements on heart seemed independent of hypoglycemic effects.

### *ASMase*^*Myh6KO*^* mice reduces NAPDH oxidase activity and apoptosis in HFD mice*

**Fig. 7:**
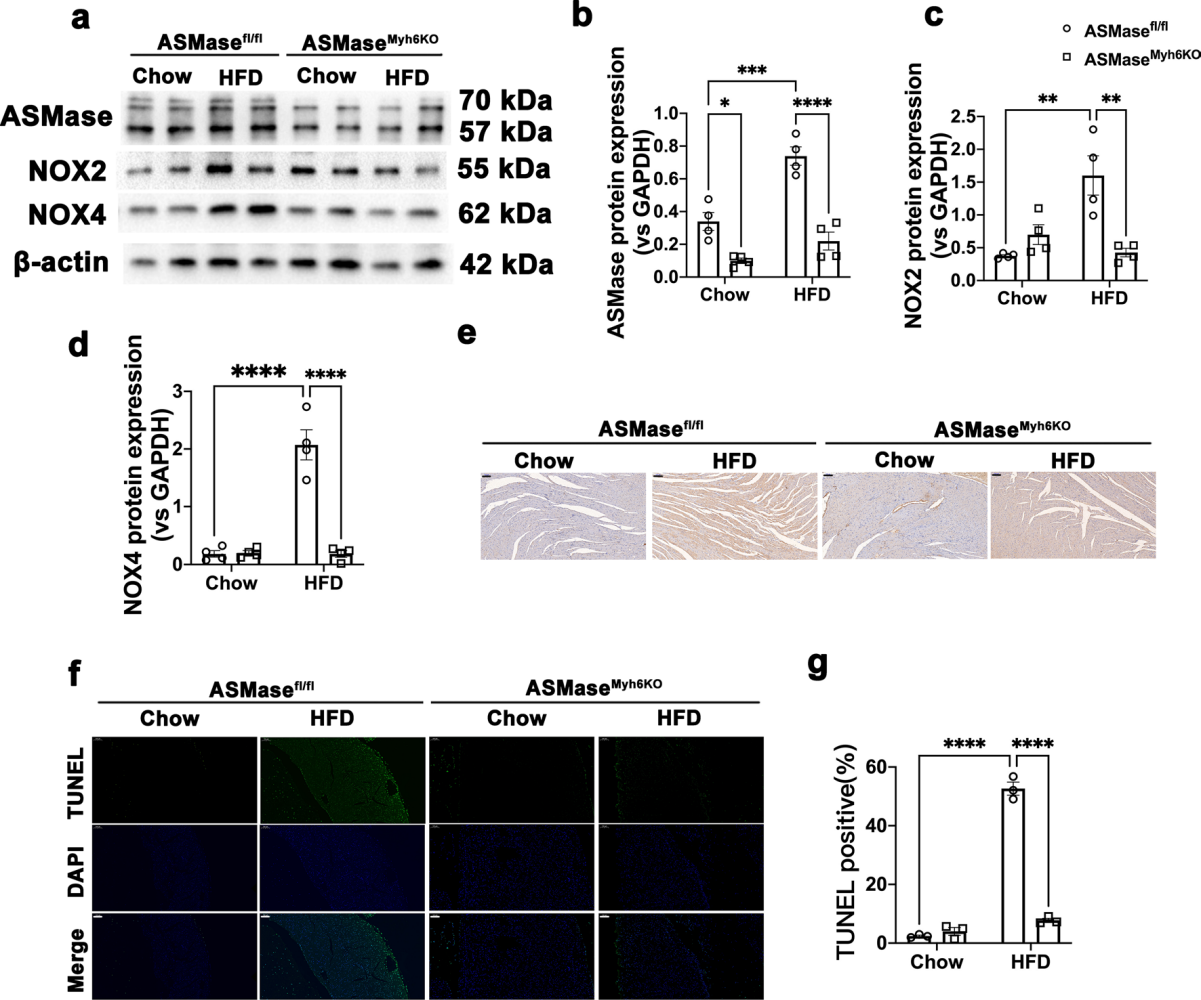
ASMase deficiency reduces NAPDH oxidase activity and apoptosis in HFD mice. **a**–**d** Representative Western blot images and densitometric analysis of ASMase, NOX2 and NOX4 protein levels in ASMase^Myh6KO^ and control mice treated with or without HFD. **e** Representative immumohistochemical staining of ceramide in myocardium of mice (scale bar:100 μm). **f**–**g** Representative fluorescence images and quantitative analysis of TUNEL staining (scale bar:100 μm). Data were presented as Mean ± SEM, n = 4. ^*^P < 0.05, ^**^P < 0.01, ^***^P < 0.001, ^****^P < 0.0001

Given the observation that ASMase^Myh6KO^ mice are protected from experimental cardiomyopathy and demonstrated in cellular experiments that ASMase was responsive to cardiac hypertrophy through NADPH oxidases activations and apoptosis, we hypothesized that the protective effects of ASMase deficiency were attributed to blockade of oxidative stress and apoptosis mediated by NAPDH oxidases. To verify this hypothesis, we accessed NADPH oxidase 2 and 4 expressions and apoptosis in ASMase-deficient mice induced by HFD. We found that HFD-induced ASMase upregulation was significantly blocked by ASMase knockout, like the findings *invitro* using siRNA (Fig. 7a–b). In agreement with the inhibition of ASMase, the NOX2 and NOX4 expressions elicited by HFD were both significantly decreased in the hearts from ASMase-deficient mice (Fig. 7a, c–d). Again, the immumohistochemical staining data mirrored that ceramide content tended to decrease in HFD ASMase-knockdown hearts, however, the decrease did not reach statistical significance and higher than control groups (Fig. 7e), suggesting that ceramide is not too affected by ASMase knockdown. However, the deficiency of ASMase still blocked myocardial apoptosis in HFD-treated mouse heart (Fig. 7f–g), indicating ASMase is independent of ceramide. Together, our results strongly suggest that therapeutic strategies targeted to ASMase-mediated activation might be beneficial in attenuating oxidative stress, apoptosis and cardiomyopathy associated with obesity, type 2 diabetes, and insulin resistance.

## Discussion

The key findings of this study are: (1) ASMase upstream of NOX4 is responsible for positive regulation of oxidative stress-related apoptosis; (2) Cardiac ASMase upregulation caused cardiomyocytes remodeling, dysfunction, and apoptosis in the HFD-fed mice; (3) NOX 4 activation is required for the effect of ASMase in the face of metabolic stress. At least, deletion of ASMase alleviates apoptosis and restores cardiac function, revealing a potential new strategy to negate cardiomyopathy from obesity and diabetes.

A growing body of clinical data suggesting that ceramides, in particular Cer-16 (ceramide with acylated palmitic acid) are associated with both higher diabetes risk and CVD risk after diabetes onset [12]. However, several conflicting results were reported by different trials. For instance, cell and animal studies have suggested that Cer-16 promotes apoptosis, while longer chain ceramides, such as Cer-20, -22, and -24, appear to prevent apoptosis [13–15]. Wang C demonstrated that ceramide accumulation caused by smpd1/2 upregulation plays a crucial role in alcoholic cardiomyopathy [16], while other scholars obliterated ceramides by specific deletion of serine palmitoyltransferase subunit 2 (a de novo synthase of ceramides) in cardiomyocytes also led to cardiac dysfunction [17]. These conflicting results promote us to suspect that the adverse effect of ceramides on cardiomyocytes may be regulated or impacted by some other factors, such as ASMase, the key enzyme in the hydrolysis/salvage pathway to produce ceramides primarily catalyzing sphingomyelin. Previous work has shown some mechanistic insight into the role of ASMase in mediating cardiovascular diseases. ASMase mediated TNF-α-induced endothelial dysfunction through inhibiting eNOS phosphorylation and activating of MAPK signaling, which were restored by its inhibitor, amitriptyline [18]*.* Moreover, targeting the ASMase pathway is protected from hypertensive vascular damage [11]. In our present study, HFD-induced apoptosis and consequential cardiac dysfunction were completely prevented by knockdown of ASMase or imipramine treatment. It is generally believed that hyperglycemia, hyperlipidemia occurring with obesity may increase cardiac lipid uptake and formation of ceramides, DAG and other lipids, which initially promotes cardiomyopathy. Actually, cardiac ceramide content tended to decrease in ASMase^Myh6KO^ mice and imipramine treated mice as compared to control group, however, this decrease did not reach to the normal levels, thus indicating that HFD-induced cardiomyopathy caused by ASMase overexpression, at least in the context of cardiac lipid overload, is not entirely dependent on ceramide generation. This data is in accordance with a previous study evidenced that targeting ceramide accumulation in the ischemic heart may not be a beneficial treatment strategy [19].

ASMase can be activated by pathological factors, such as cytotoxic agents, proinflammatory cytokines, lipopolysaccharide, PA and others [20]. To simulate the in vivo experiment of metabolic disturbance, H9c2 cardiomyocytes were cultured under metabolic stress by administration of high glucose, palmitic acid and C16-cemamide.Consistent with in vivo observation, ASMase was activated by co-incubation of the high glucose and PA as expected, cardiac hypertrophy has also been observed as shown by increased cardiomyocytes sizes and transcriptional levels of *Nppb* and *Myh 7* genes, which were considered as pro-hypertrophic markers. Unexpectedly, a similar effect on ASMase activation was also caused by addition of C16-ceramide, but cardiac hypertrophy was not happened, suggesting that ceramide can activate ASMase in a positive feedback manner to form a vicious cycle. It is noteworthy that both high glucose and PA and C16-Cer increased proportion of TUNEL-positive cardiomyocytes and cleaved-caspase 3 protein expression, which indicates the occurrence of apoptosis. This hints that C16-Cer mediated cardiac injury and cell death are not associated with cardiac hypertrophy. The lipotoxicity of ceramides is due to the side chain length, as those composed of C16:0 or C18:0 side chain is toxic [21]. Bekhite M. et al. reported that the long-chain ceramide accumulation led to mitochondrial oxidative stress and consequently mitochondrial damage and dysfunction in cardiomyocytes model [22]. We speculated that this direct lipotoxic effect on mitochondria may evoked cell apoptosis without mediating hypertrophic response. Nevertheless, the exact mechanism of accounting for this difference between high glucose, PA and C16-Cer was not addressed in this study and further works are needed to clarify this point.

Increased lipid uptake and metabolites formation can activate membrane bound NADPH oxidases (NOXs) to generate ROS. At early stage, the increasing of cytosolic ROS enhances fatty acid oxidation to generate more ATP as an adaptive reaction for energy balance. Continuing metabolic stress leads to compromised fatty acids and glucose oxidation [23]. In this case, aberrant lipids accumulation in the cytosol will further augment NOXs-generated ROS production, eventually damaging mitochondria and consequent cardiac injury. Using animal models with excess dietary fat, our present study demonstrated that ASMase is likely a culprit for high glucose, PA and C16-Cer stimulated ROS production by regulation of NADPH oxidases, mainly is NOX4.NOX4 is a major source of cytosolic ROS, which initiate oxidative stress in diabetes [24]. In our study, we documented that ASMase increased ROS production by stimulating NOX4 expression, which triggered apoptosis. This effect was completely prevented by ASMase inhibitor or knockout of ASMase, suggesting that ASMase, per se, may produce a cardiac damage effect by directly promoting NOX4 activation. NOX4 inhibition by apocynin effectively prevented cardiomyocytes hypertrophy as well as apoptosis induced by ASMase. Di Pietro P and colleagues demonstrated that ASMase can mediate NOX2 activation and required the action of endogenous sphingosine-1-phosphate (S1P) to induced endothelial dysfunction and vascular damage in hypertension [11]. However, we did not find any noticeable change of NOX2 expression in our present research. This difference may be due to the distinct disease model and tissue specificity. Furthermore, the mRNA transcriptional levels of major NADPH oxidases, such as NOX4, NOX2 and NOX1, were not differ statistically across groups, suggesting that ASMase regulates NOX4 at the posttranscriptional level.

We further verified in vivo that defective of cardiac ASMase restored HFD-induced cardiomyopathy in mice, manifesting improved cardiac function, less hypertrophy, fibrosis, and apoptosis. The beneficial effects of ASMase deletion in DCM can be attributed to the antioxidant consequences as NOX 4 expression was suppressed in ASMase^Myh6KO^ mice. In comparison, ceramide reduction was not so obvious. This suggest that ASMase is involved in a direct pathway that contribute to HFD-induced cardiomyopathy.

## Supplementary Information

**Additional file 1: Figure** **S1.** Genotyping of cardiomyocyte-specific ASMase-knockout mice. Representative genotyping results for the ASMase^Myh6KO^ and control littermates. On introduction of cre-recombinase (Myh6-cre), exon 2 of the ASMase gene was excised specifically in cardiomyocytes, allowing for generation of selective ASMase knockout mice (ASMase^Myh6KO^). In the schematic diagram, pups 1, 2, 3 and 6 were identified as ASMase^Myh6KO^ [ASMase^fl/fl^ with cre recombinase (Cre+/0)], and pups 4 and 5were control littermates [ASMase^fl/fl^ without cre recombinase (Cre0/0)].

**Additional file 2: Table S1.** Description of the primers used in this study. NOX1: NADPH oxidase 1; NOX2: NADPH oxidase 2; NOX4: NADPH oxidase 4; Nppb: natriuretic peptide B; Myh7: Myosin Heavy Chain 7; GAPDH: glyceraldehyde-3-phosphate dehydrogenase.

## Data Availability

All data generated or analysed during this study are included in this published article and its supplementary information files. The datasets used and/or analyzed during the current study are available from the corresponding author on reasonable request.
