# Supplementary material for: Acid sphingomyelinase promotes diabetic cardiomyopathy via NADPH oxidase 4 mediated apoptosis
Source: Cardiovasc Diabetol. 2023 Feb 2;22:25. doi: 10.1186/s12933-023-01747-1 (PMC9896821; doi:10.1186/s12933-023-01747-1)
Supplement: Supplementary file 1 — Additional file 1: Figure S1. Genotyping of cardiomyocyte-specific ASMase-knockout mice. Representative genotyping results for the ASMaseMyh6KO and control littermates. On introduction of cre-recombinase (Myh6-cre), exon 2 of the ASMase gene was excised specifically in cardiomyocytes, allowing for generation of selective ASMase knockout mice (ASMaseMyh6KO). In the schematic diagram, pups 1, 2, 3 and 6 were identified as ASMaseMyh6KO [ASMasefl/fl with cre recombinase (Cre+/0)], and pups 4 and 5were control littermates [ASMasefl/fl without cre recombinase (Cre0/0)]. [file 12933_2023_1747_MOESM1_ESM.docx]

**Supplemental figure**


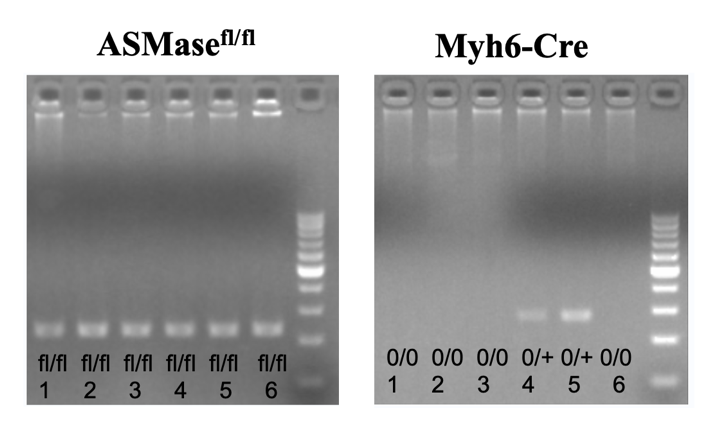


#1,2,3,6: ASMase^fl/fl^; #4,5: ASMase^Myh6KO^

**Supplemental figure** **S1.** **Genotyping of cardiomyocyte-specific ASMase-knockout mice.** Representative genotyping results for the ASMase^Myh6KO^ and control littermates. On introduction of cre-recombinase (Myh6-cre), exon 2 of the ASMase gene was excised specifically in cardiomyocytes, allowing for generation of selective ASMase knockout mice (ASMase^Myh6KO^). In the schematic diagram, pups 1, 2, 3 and 6 were identified as ASMase^Myh6KO^ [ASMase^fl/fl^ with cre recombinase (Cre+/0)], and pups 4 and 5were control littermates [ASMase^fl/fl^ without cre recombinase (Cre0/0)].
