# Supplementary material for: Acid sphingomyelinase promotes diabetic cardiomyopathy via NADPH oxidase 4 mediated apoptosis
Source: Cardiovasc Diabetol. 2023 Feb 2;22:25. doi: 10.1186/s12933-023-01747-1 (PMC9896821; doi:10.1186/s12933-023-01747-1)
Supplement: Supplementary file 2 — Additional file 2: Table S1. Description of the primers used in this study. NOX1: NADPH oxidase 1; NOX2: NADPH oxidase 2; NOX4: NADPH oxidase 4; Nppb: natriuretic peptide B; Myh7: Myosin Heavy Chain 7; GAPDH: glyceraldehyde-3-phosphate dehydrogenase. [file 12933_2023_1747_MOESM2_ESM.docx]

**Supplemental table**

**Additional file 2: Table S1. Description of the primers used in this study.** NOX1: NADPH oxidase 1; NOX2: NADPH oxidase 2; NOX4: NADPH oxidase 4; Nppb: natriuretic peptide B; Myh7: Myosin Heavy Chain 7; GAPDH: glyceraldehyde-3-phosphate dehydrogenase.

Supplemental table S1: Primer List

| **#** | **Names of Primer** | **Sequence of Primer** |
| --- | --- | --- |
| 1 | *NOX1(rat)* *Forward* | 5’- TTCCCTGGAACAAGAGATGG -3’ |
| 2 | *NOX1(rat)* *Reverse* | 5’- GACGTCAGTGGCTCTGTCAA -3’ |
| 3 | *NOX2 (rat) Forward* | 5’- ACCAAGGTGGTCACTCATCC -3’ |
| 4 | *NOX2 (rat) Reverse* | 5’- ACAATGCGGATATGGATGCT -3’ |
| 5 | *NOX4 (rat) Forward* | 5’- ACAGTCCTGGCTTACCTTCG -3’ |
| 6 | *NOX4 (rat) Reverse* | 5’- CTGAGAAGTTCAGGGCGTTC -3’ |
| 7 | *Nppb (rat) Forward* | 5’-TTTGGGCAGAAGATAGACCG-3’ |
| 8 | *Nppb (rat) Reverse* | 5’-AGAAGAGCCGCAGGCAGAG-3’ |
| 9 | *Myh7 (rat) Forward* | 5'-CGGTGACTGTGAAGGAGGAC-3' |
| 10 | *Myh7* *(rat) Reverse* | 5'-TGAGATTGTAGAGCACAGCCG-3' |
| 11 | *Gapdh (rat) Forward* | 5’-ATGGGAAGCTGGTCATCAAC-3’ |
| 12 | *Gapdh (rat) Reverse* | 5’-GTGGTTCACACCCATCACAA-3’ |
